# Strategies to Modulate Specialized Metabolism in Mediterranean Crops: From Molecular Aspects to Field

**DOI:** 10.3390/ijms22062887

**Published:** 2021-03-12

**Authors:** Raffaella Balestrini, Cecilia Brunetti, Maria Cammareri, Sofia Caretto, Valeria Cavallaro, Eleonora Cominelli, Monica De Palma, Teresa Docimo, Giovanna Giovinazzo, Silvana Grandillo, Franca Locatelli, Erica Lumini, Dario Paolo, Cristina Patanè, Francesca Sparvoli, Marina Tucci, Elisa Zampieri

**Affiliations:** 1 National Research Council (CNR)-Institute of Sustainable Plant Protection (IPSP), Viale Mattioli 25 and Strada delle Cacce 73, 10125 and 10135 Torino, Via Madonna del Piano 10, 50019 Sesto Fiorentino, Italy; cecilia.brunetti@ipsp.cnr.it (C.B.); erica.lumini@ipsp.cnr.it (E.L.); elisa.zampieri@ipsp.cnr.it (E.Z.); 2 CNR-Institute of Bioscience and Bioresources (IBBR), Via Università 133, 80055 Portici, Italy; maria.cammareri@ibbr.cnr.it (M.C.); monica.depalma@ibbr.cnr.it (M.D.P.); teresa.docimo@ibbr.cnr.it (T.D.); silvana.grandillo@ibbr.cnr.it (S.G.); marina.tucci@ibbr.cnr.it (M.T.); 3 CNR-Institute of Sciences of Food Production, Via Monteroni, 73100 Lecce, Italy; sofia.caretto@ispa.cnr.it (S.C.); giovanna.giovinazzo@ispa.cnr.it (G.G.); 4 CNR-Institute of Bioeconomy (IBE), Via Paolo Gaifami, 18, 95126 Catania, Italy; valeria.cavallaro@ibe.cnr.it (V.C.); cristinamaria.patane@cnr.it (C.P.); 5 CNR-Institute of Agricultural Biology and Biotechnology, Via Edoardo Bassini 15, 20133 Milan, Italy; cominelli@ibba.cnr.it (E.C.); locatelli@ibba.cnr.it (F.L.); d.paolo@ibba.cnr.it (D.P.); sparvoli@ibba.cnr.it (F.S.)

**Keywords:** advanced breeding, arbuscular mycorrhizal fungi, biotechnological approaches, cell bio-factory, elicitation, glandular trichomes, omics, plant growth-promoting bacteria, secondary metabolites, stress response, transcriptional networks

## Abstract

Plant specialized metabolites (SMs) play an important role in the interaction with the environment and are part of the plant defense response. These natural products are volatile, semi-volatile and non-volatile compounds produced from common building blocks deriving from primary metabolic pathways and rapidly evolved to allow a better adaptation of plants to environmental cues. Specialized metabolites include terpenes, flavonoids, alkaloids, glucosinolates, tannins, resins, etc. that can be used as phytochemicals, food additives, flavoring agents and pharmaceutical compounds. This review will be focused on Mediterranean crop plants as a source of SMs, with a special attention on the strategies that can be used to modulate their production, including abiotic stresses, interaction with beneficial soil microorganisms and novel genetic approaches.

## 1. Introduction

Plants are excellent chemists because of their ability to produce an outstandingly wide arsenal of not less than 5000 different metabolites [1]. However, the real extension of the diversity of natural compounds is still largely unknown, since only a small portion of the plant world has so far been explored from a chemical point of view. These natural products are volatile, semi-volatile and non-volatile compounds derived from common building blocks produced through primary metabolic pathways, such as amino acids, isoprenoids, nucleotides and sugars [2]. They have long been considered waste products of the metabolism, and the name “secondary metabolites” derives from the initial observations that, in contrast to primary metabolites, they are not directly involved in growth and reproduction [3]. However, they should be more appropriately referred to as specialized metabolites (SMs). Differently from the evolutionary constrained primary metabolites, SMs rapidly evolve in order to allow a better adaptation of plants to environmental cues [4,5]. In plants, these chemo-diverse molecules add color, taste and odor but can be also toxic to other organisms and deter herbivores and pathogens, protect from UV light, cold and drought [6] or be attractive to fascinate pollinators and disperse seeds [7]. There are three major groups of SMs in plants based on their biosynthetic pathways, i.e., nitrogen-containing compounds (cyanogenic glycosides, alkaloids and glucosinolates), phenolic compounds (phenylpropanoids) and terpenes (isoprenoids) [8]. These few main carbon backbones are further modified through chemical reactions such as glycosylation, acylation, methylation, hydroxylation, prenylation, etc., generating a wealth of phytochemicals with diverse biological properties and interaction capacities. These specific enzymatic decorations generally occur in phylogenetically restricted groups of plant species, in particular developmental stages, in specific tissues or cell types or following favorable or unpleasant interactions [9,10]. As an example of such specialization, tropane alkaloids are biosynthesized in roots to be then translocated to leaves, as in the Solanaceae, or biosynthesized and accumulated in leaves, as in the Erythroxylaceae [11]. Roots can also produce specialized molecules such as benzoxazinoids, naphthoquinones and strigolactones, which are secreted in the environment and are responsible for important allelopathic interactions. SMs are considered essential for the defense of plants from biotic and abiotic stresses [12,13], often exacerbated by global climate change [14]. Climate change involves a general rise of atmospheric CO_2_, temperature and drought, even in those regions classified as sub-humid up to a few decades ago. Under such stressing environmental conditions, plants adjust their metabolism towards an energy-costly production of SMs, in order to improve their acclimation and survival strategies [15,16]. Besides their role in plant physiology, SMs are also of outstanding interest for other reasons. Humans have in fact benefitted from SMs in agriculture, medicine, production of flavors and perfumes, etc. Most of the SMs present in plants, especially in fruits, have antioxidant effects; therefore, their consumption in foods may contribute to human health, since they provide protection against the constant formation of reactive oxygen species (ROS) which are powerful oxidants [17]. Pharmaceutical organic chemists have been long investigating the chemical properties of these phytochemicals, for their potential application as fibers, oils, flavoring agents, dyes and drugs, as well as antibiotics and agrochemical substitutes. Indeed, most of these molecules are produced in plants for their strong anti-herbivore activity, as well as protection against pathogens and competing plant species, having a high potential as bio-pesticides and herbicides [18].

**Figure 1:**
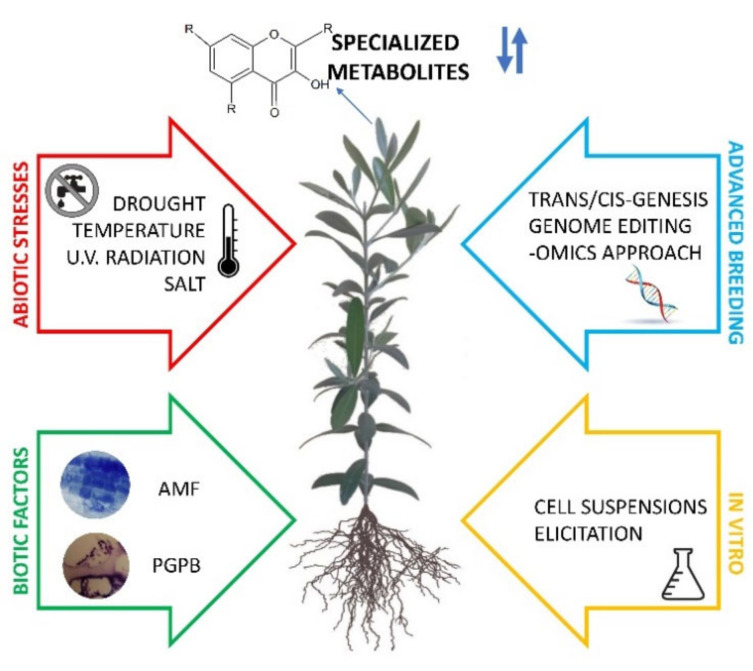
Scheme representing the diverse factors (natural, on the left, and artificial, on the right part) that can modulate specialized metabolites in plants: abiotic stresses as drought, salt, UV radiation and temperature; microorganisms (i.e., biotic factors) such as plant growth-promoting bacteria—PGPB and arbuscular mycorrhizal fungi—AMF; in vitro systems; advanced breeding techniques based on trans/cis-genesis, genome editing and -omics approaches.

The wide range of applications of SMs and the recognition of their biological properties have supported the interest towards these bioactive compounds by researchers and the development of crops for bioactive compounds production by agronomists [17]. In this review, attention will be focused on Mediterranean crop plants as a source of SMs, with a glimpse on the strategies that can be used to modulate their production (Figure 1).

## 2. Specialized Metabolite Production in Response to Abiotic and Biotic Factors

### 2.1. Specialized Metabolites in Mediterranean Crops in Response to Abiotic Stresses and Agronomic Management

In Mediterranean regions, plants are exposed to multiple interacting environmental stressors such as drought, heat, salinity and excess UV radiation. Environmental stress factors can weaken important physiological functions of the plant and severely affect crop yields. Under these stressful conditions, plants address their metabolism and gene expression towards physiological and morphological adaptation and the biosynthesis of defensive SMs rather than nitrogen-based compounds. This “metabolic plasticity is costly, as fresh assimilated carbon and energy are diverted from growth but may represent a key determinant for the survival of plants in harsh environments, such as diverse regions in the Mediterranean area. Overall, factors that determine alterations in SMs are mainly environmental and genetic. Among them, environmental factors are the key determinants for the fluctuations in plant SMs [19]. Indeed, genetic factor influence has been documented for example in wheat where a reduction of the polyphenolic richness in modern cultivars, as compared to the old varieties, was observed [20,21]. These results, however, may be also indirectly connected to environmental conditions, since in the past the selection pressure was addressed more to plant adaptability to environmental stresses than to high productivity as in modern cultivars. A detailed but not exhaustive overview on the effects of some main environmental stresses on SM induction in some important Mediterranean crops are thereafter discussed.

#### 2.1.1. Main Environmental Stresses and Specialized Metabolites in Different Crops

*High radiation/UV Stress*. Plants require appropriate intensity of light for photosynthesis, which in turn influences the quality and accumulation of SMs. Therefore, the literature indicates the conspicuous effect of photoperiod and light intensity on the biosynthesis and storage of SMs [19]. Lettuce, spinach, brassica, wheat, strawberry, tomato among the horticultural crops and apple and grapevine showed increases in polyphenols under UV or light stress [8,22,23].

*Heat/cold stress*. The biosynthesis of SMs is also correlated with heat stress in plants [19]. Three literature surveys [8,22,23] indicate that high-temperature stress usually increased the production of phenolics in some crops such as lettuce, spinach, tomato, watermelon, lentil and sorghum. It was suggested that thermal stress induced the production of phenolics and thereby led to thermal tolerance in plants. In sorghum, authors correlated the rich phenolic profile of the dark seeded genotypes with greater temperature tolerance. It was also ascertained a marked variation in the response of plant species to different temperature ranges. Low temperature also caused a conspicuous decrease in the biosynthesis and storage of SMs [19] in different crops while an increase was reported in pepper [8,24]. In *Vitis vinifera*, it was proposed that tolerant varieties possess higher reducing power, antioxidant activity and phenolic contents. In this species, cold stress also resulted in a marked decrease in the concentration of phenolics (ferulic acid, p-coumaric acid, caffeic acid, and particularly caffeic acid derivatives) and antioxidant activity. A similar decrease was reported for rosmarinic acid content in spearmint under heat stress [22].

*Salt Stress*. Plant SMs may undergo increase or decrease in their concentration in response to salinity-induced osmotic stress or specific ion toxicity. Salinity increased SMs and antioxidant potential in cotton (namely tannic acid, flavonoids and gossypol), in safflower plants growing under <100 mM of NaCl (flavonoids), in buckwheat (carotenoids, phenolic compounds and antioxidant activity), in artichoke and cultivated cardoon (flavonoids and chlorogenic acid) in response to moderate and high levels of salinity [25]. Increase in SMs in response to salt stress has been reported also in *Rosmarinus officinalis*, maize, wheat, pea, strawberry, spinach, *Brassica oleracea*, barley, basil, *Matricaria chamomilla* and some other spontaneous herbs (*Mentha pulegium*, *Nigella sativa*) [8,26]. Differently, a decrease in SMs was observed in lentil and *Phaseolus vulgaris*. The increase in SMs (i.e., polyphenols), induced by salinity stress, involves an improvement in the antioxidant activity in some important crops (wheat, buckwheat, artichoke, rice and cultivated cardoon), thus improving their nutritional quality [8,22,23,25]. Salt stress also resulted in a significant rise in alkaloid concentration in *Achillea fragrantissima*, *Catharanthus roseus* and *Solanum nigrum*. There are contrasting reports on the production of essential oils in aromatic plants upon salinity stress, and the levels of SMs also vary in response to the availability of different nutrients [19].

*Drought Stress*. Several SMs produced in plants are helpful in the induction of drought tolerance [19]. The rise in endogenous levels of plant SMs (phenolics) in response to drought stress was recorded in *Brassica napus*, *Lactuca sativa*, *Cucumis sativus*, *Thymus vulgaris* and many medicinal plants [19,27,28]. In grapevine and *Triticum aestivum*, the concentration in phenolic compounds markedly increased in plants subjected to drought stress. In maize and in triticale, under polyethylene-glycol induced stress, the accumulation of ferulic acid and total phenolics was observed in drought-tolerant genotypes. Thus, in these species, the accumulation of phenolics and ferulic acid could be used as a selection criterion to screen plants for drought resistance [8,23].

#### 2.1.2. Two Case-Studies: Tomato and Olive

Tomato (*Solanum lycopersicum* L. *syn*. *Lycopersicon esclulentum* Mill.) is one of the most relevant Mediterranean crops, and it is a good source of natural antioxidants, such as ascorbic acid, carotenoids, polyphenols, etc., thus playing an important role in human nutrition and in prevention of cancer and cardiovascular diseases [29,30]. The content of these constituents has been evidenced to largely depend on both the environmental and agronomic factors such as cultivation area, cultivar, irrigation and fertilization.

Interestingly, there is evidence that tomato fruits produced in Mediterranean areas are richer in phenols than those produced in Northern Europe [31]. Siracusa et al. [32], working on some local Mediterranean landraces of long shelf-life tomato cultivated in a typical semi-arid environment under no water supply, observed greater contents in total phenols (0.09 to 0.21 mg g^−1^ of fresh weight), up to ten-fold higher than those measured in the commercial cultivar used as control (<0.030 mg g^−1^). The greatest phenol contents in long shelf-life landraces were explained as a result of environmental pressure, which exerted a natural selection towards those types with relevant phenolic biosynthesis, being these landraces traditionally cultivated under no water supply.

Patanè et al. [33], working on long-shelf-life tomato landraces cultivated in both greenhouse and open field conditions in Southern Italy, reported a more pronounced accumulation of phenolics in open field fruits, as a result of fruit exposition to solar UV radiation and high temperatures (up to 32 °C) during ripening. The authors explained the lower phenolic content in greenhouse fruits as a possible result of UV-exclusion under PVC cover and low temperatures occurring in late wintertime inside the unheated greenhouse. Similar promoting effects of high levels of irradiance in open fields were reported by the same authors on ascorbate accumulation in tomato fruits (up to +65%). An increase of phenolics in tomatoes grown under temperatures approaching 35 °C was also observed by Rivero et al. [34], as a mechanism of adaptation against heat stress. These authors explained this result as an activation of the enzyme PAL under high temperatures, which induces a rise in phenolic content of fruits [22].

Beside genotype and climatic factors, the agronomic management, mostly irrigation, greatly affects the content of SMs in the product. In processing tomatoes cultivated in a typical semi-arid area of Southern Italy, an increased content in ascorbic acid and total phenolics, has been reported in response to moderate or severe water stress conditions [35,36]. These results reveal that it is possible to manage water stress through the application of water-saving irrigation strategies (e.g., deficit irrigation) in order to promote the nutritional tomato properties, in terms of SM content.

Several studies also revealed a stimulating effect of salinity on ascorbic acid in tomato fruits [22]. Sgherri et al. [37] explained an increase in ascorbic acid content in tomato under salinity stress with a de novo synthesis beside an enhanced ascorbate recycling via recycling enzymes coupled with the reducing compound dihydrolipoic acid. A stimulating effect of different environmental stresses has been demonstrated also on tocopherol (vitamin E) content of tomato fruits [38].

Olive (*Olea europaea* L.) has been exploited for the polyphenolic content of its fruits and oil; however, a high and rich content of polyphenols has been recently recognized also in olive leaves by Kulak and Cetinkaya [39]. This high content in polyphenols confers resistance against abiotic and biotic stressors. This raw material is actually being studied as a source of useful bioactive compounds with antioxidants (mainly due to oleuropein), antimicrobial and human health properties [40]. In the Mediterranean region, olive is the prevalent crop as a source of oil. Olive oil includes high amounts of SMs, namely, α-tocopherol, oleuropein, hydroxytyrosol, tyrosol, caffeic acid, ferulic acid, *p*-coumaric, vanillic acid, apigenin, luteolin, pinoresinol, 1-acetoxypinoresinol, oleocanthal and oleacein, most of which are phenolic compounds. The quality and subsequently aromatic value or bio-efficacy of the oil or olive fruits have been well documented to be related to the content and pattern of phenolic compounds of the fruit.

The quantity and quality of the SMs in olive parts are not stable. In short, multivariate interactions of endogenous (cultivar, plant age, crop developmental stage and alternate bearing phenomenon) and exogenous factors (water and salinity stress, environment, agricultural practices, etc.) are predictive factors of the amount of phenols present in olive leaves, fruits and oil [39]. Guo et al. [41] suggested that the optimal harvest time for each olive variety permits to obtain the highest phenolic content in oil. In fact, increased quantities of phenolic compounds have been recorded from the green to the spotted stages of maturation, and then, they decrease until maturity.

In detail, as concerns the effect of water limitation, it triggered the biosynthesis of phenolic compounds, suggesting the possible antioxidant roles of phenolics in the leaves. Relevant increases in flavanols in the leaves, according to the genotype (Kilis Yağlık), the lack of irrigation and the developmental stages of olive, have been ascertained. The authors hypothesize that the increase in leaf biosynthesis of flavonoids in summer could be related to their role in the protection against stress induced by UV irradiations. In fact, the flavonoid functions against UV irradiation stress have been well documented [42].

As concerns salinity, changes in phenolic compounds of *O. europaea* cultivars contrasting in salt tolerance, have been reported. Phenolic content in new leaves of salt-sensitive cultivars remained stable at increasing salinity levels whereas salt-tolerant cultivars exhibited greater phenolic contents, which raised with the increase in salt stress in new and old leaves. Within the quantified phenolic compounds, oleuropein was reported to be the major compound in both leaves and roots, suggesting the possible protective role against salt stress [39].

Ecological variability in the phenolic compounds of *O. europaea* leaves from natural habitats and cultivated conditions was also reported [39]. Interesting output of the research was that samples from cultivated plants exhibited higher total amount of phenols and flavonoids, but the antioxidant activity of the extracts was higher in samples from natural habitats.

### 2.2. Impact of Beneficial Microbes to Enhance the Content of Specialized Metabolites

Production and accumulation of SMs often occur when plants are subjected to various stresses, elicitors or signal molecules [43]. Some major biotic elicitors with a role in the production of SMs in plants are arbuscular mycorrhizal fungi (AMF) and plant growth-promoting bacteria (PGPB) [44,45]. The use of AMF during cultivation is a promising possibility to increase biomass and essential oil yield of medicinal, spicy, aromatic plants through less aggressive agronomic practices that stimulate plant growth, without compromising the quality of the merchandised product. The application of AMF in these cultivations has been already reported to improve plant growth and development as well as the tolerance to abiotic and biotic stressors [46,47,48]. It is already known that AMF can influence the plant specialized metabolic pathways [49], and there is some evidence that AM fungal colonization increases phenolic content and resistance to oxidative stress in fruit tissues [49,50,51,52]. In this context, it has been proposed that AMF, either naturally occurring or inoculated, may be a sustainable approach to alleviate malnutrition and supplementing human health as part of the “second green revolution” [53]. PGPB can increase plant growth, improving plant nutrition and supporting plant development under natural or stressed conditions. Indeed, beneficial bacterial inoculants may be recognized as a potential threat by the plant and induce the activation of plant defense mechanisms leading to the biosynthesis of resistance compounds [45]. Several studies confirmed that different bacterial species living on and inside the root system are beneficial for plant growth, yield and crop quality, including *Pseudomonas*, *Bacillus*, *Azotobacter* and *Azospirillum* spp. capable of inducing SM production in different aromatic plants such as *Salvia officinalis*, *Origanum* × *majoricum*, *Origanum majorana* and *Ocimum basilicum* [45]. In addition, enhanced production of apigenin-7-O-glucoside in chamomile was triggered by a treatment with two bacterial strains such as *Bacillus subtilis* Co1-6 and *Paenibacillus polymyxa* Mc5Re-14 [54], while *Chaetomium globosum* D38 promoted bioactive constituent accumulation and root production in *Salvia miltiorrhiza*, increasing the content in tanshinones, a molecule recently utilized in the treatment of several cardiovascular diseases [55].

In the last ten years, several research groups focused the attention to evaluate the impact of AMF on the SM production in different horticultural crop species as well as in aromatic and medicinal plants. Although a positive impact on both yield and quality has been found [56,57,58], this is not always reported [59], probably depending on the genotypes and the cultivation conditions. In this paragraph, some examples of these researches have been reported, with the aim to underline the importance of the AM symbiosis in the cultivation of these plant species.

Already in 2013, Lingua and colleagues [52] showed that inoculation with AMF and/or with selected *Pseudomonas* strains increased anthocyanin concentration in strawberry (*Fragaria* × *ananassa* var. Selva) fruits under conditions of reduced fertilization. An increase in strawberry fruit anthocyanin content has been also reported after inoculation with AMF communities from soils collected in reference-sites in the cultivation of this species [60]. A higher concentration of sugars and ascorbic and folic acid in comparison with fruits of non-inoculated plants has been also reported after an inoculation with a mixed inoculum containing both AMF and two PGPB strains [56].

However, antioxidant content is influenced by cultivars, growing environments, production methods, harvest time, storage and transport [61]. Among the soil management practices, several studies have been focused to verify the impact of the AMF inoculation, alone or in combination with PGPB, on tomato performance and fruit nutritional compounds [61,62,63,64,65,66]. Transcriptomics of fruits collected from inoculated plants has been also performed [67]. Overall, the interaction of tomato plants with AMF enhances the nutritional quality by increasing the contents of citric acid, carotenoids and certain amino acids as well as the antioxidant capacity of the fruits. In one of the first work dedicated to verifying the impact of arbuscular mycorrhiza (AM) symbiosis on tomato fruits, Giovannetti et al. [63] showed that the AM symbiosis, in addition to positively affecting the tomato plant growth and mineral nutrient content, also enhanced the nutritional and nutraceutical value of tomato fruits by modifications of plant specialized metabolism that led to increased levels of lycopene in fruits from AM-colonized plants with respect to control ones. An AMF-mediated increase in lycopene content of tomato fruits has been also observed in a recent study [66] focused on cherry tomato landraces belonging to the Protected Designation of Origin (PDO) “Pomodorino del Piennolo del Vesuvio” (PPV), which represents one of the most typical agricultural products of the Campania region (Southern Italy). Focusing on both the yellow-pigmented type (“Giagiù”) and red-pigmented type (“Lucariello”) tomatoes, it was shown that AMF inoculation positively influenced the content of molecules with a role in both human health and shelf life of tomato fruits, with a genotype-specific response. A significant increase in lycopene, total ascorbic acid (TAA), alanine, gamma-aminobutyric acid (GABA) and branched-chain amino acids (BCAA) has been in fact found in “Lucariello” local variety, as well as an increase in calcium, zinc, ASP, GABA and the essential amino acids arginine and lysine in “Giagiù” genotype. Schubert et al. [59] focused instead on fruit gene expression and metabolite levels in industrialized tomatoes whose production faces a decrease in flavors and nutritional value due to conventional breeding. It is worth noting that red fruits from mycorrhizal plants showed an increased level of carotenoids in comparison to those from non-mycorrhizal plants, although an effect on yield was not observed. The effect was also reported by Hart et al. [64] on a different tomato cultivar, suggesting both a direct upregulation of the carotenoid pathway due to lower carbohydrate levels and an indirect upregulation of this pathway by acting on the root hormonal levels (e.g., stimulating ABA biosynthesis that shares the same metabolic pathway of carotenoids). These authors also showed an increase in volatile organic compounds (VOCs) derived from phenylalanine in the fruits of inoculated tomatoes with five volatile compounds that were significantly higher in AM-plants compared with non-AM controls [64]. Since phenylalanine is a key precursor of the phenylpropanoid pathway, which produces several antioxidant molecules such as flavonols and caffeic acid derivatives, further studies on AMF inoculation on tomatoes cultivars could be important to investigate the antioxidant potential of the fruits.

The same positive results have been obtained on melon (*Cucumis melo*). In comparison to controls, pulp of mycorrhizal plants contained more sugars and carotenoids [68], suggesting that AM inoculation significantly improves fruit quality in the field, under commercial production conditions.

AMF are naturally associated with grapevines, providing some benefits to the plant, although the effects are dependent on many factors, including variety [69,70]. It has been observed that the association of *V. vinifera* L. *cv*. Tempranillo, which is cultivated for its wine of high quality, with AMF led to a modification in the profile of metabolites in Tempranillo berries, especially those of the primary compounds. The levels of glucose and amino acids clearly increased in berries, which may affect the aromatic characteristics of wines, while the total amount and the profiles of anthocyanins and flavonols have not been strongly affected by the AM symbiosis [71]. Moreover, Antolin et al. [69] evaluated the fruit quality of eight old grapevine varieties recovered in Navarre (northeastern of the Iberian Peninsula), colonized or not with AMFs. In some varieties, AM symbiosis improved berry traits by enhancing the concentrations of soluble solids and anthocyanins. In others, berry color, total phenolic and anthocyanin content decreased in inoculated plants, suggesting that intraspecific diversity of old grapevines could include a different capacity to respond to AM symbiosis.

In the last years, several studies have been dedicated to verifying the impact of inoculation with AMF on aromatic [72,73] and medicinal plants [74,75], mainly focusing on the impact of AMF on phenols and essential oils. In *Melissa officinalis* [76], the highest essential oil contents were observed in treatments with AMF and manure, and inoculation with AMF—without/with organic fertilizer—had positive influence on the content of citral (geranial + neral), phenols and flavonoids; in *Artemisia dracunculus* (tarragon), *Lavandula angustifolia* (lavender) and *Hyssopus officinalis* (hyssop) [77], AMF stimulated a significant increase in essential oil content in the three species. The potential role of AM symbiosis to improve the SMs content has attained enormous recognition for sustainable cultivation of medicinally important crops [78]. AMF-plant symbiosis not only improves the growth and nutrients but also exerts a synergistic effect on accumulation of bioactive compounds with medicinal importance. In chamomile (*Matricaria recutita* L.) most of the identified phenolic compounds, except apigenin, occurred in larger quantities in AM-inoculated plants, differently from that observed after inoculation with bacterial strains [54]. However, no differences between inoculated and non-inoculated plants were observed in the content and composition of essential oil, whose dominant compounds were α-bisabolol, chamazulene, and β-farnesene [79]. Another study on the same plant species showed that the presence of AMF in the soil is the most influential variable in the production of capitula and essential oil of chamomile, but subtle differences on soil pH influenced these parameters [80]. Positive effects have been also reported in the coarse mint (*Plectranthus amboinicus* Lour.), which is a medicinal plant that produces essential oil [81], and in *Bituminaria bituminosa,* which is considered a source of different phytochemicals with an importance for industrial purposes, i.e., furanocoumarins and pterocarpans that are used in cosmetics and as chemotherapeutic agents. Pistelli et al. [82] have recently demonstrated that AM-inoculated leaves of *B. bituminosa* had a high amount of furanocoumarins (angelicin and psoralen) and pterocarpans (erybraedin C and bitucarpin A), important compounds for their anticancer properties [83,84]. Furthermore, in the same study, the analysis of VOCs of inoculated plants showed different chemical composition compared with non-mycorrhizal plants, supporting previous results which suggested a positive effect of mycorrhizal symbiosis on terpenoid yield [85]. As for other aspects related to AMF colonization [86,87], the increase in SM production can change depending on the relationship between the host and the AMF species. Plants of *Cynara cardunculus* L. var. *scolymus* F. accumulated more phenolic compounds and showed a higher antioxidant activity when inoculated with *Rhizophagus intraradices* compared to those associated with *Funneliformis mosseae* (formerly *Glomus mosseae*) [88] that instead concentrated more rosmarinic and caffeic acid than those colonized with *R. intraradices* [89]. The latter was also more efficient in enhancing the level of saponins in *Chlorophytum borivilianum* Santapau and Fernandes compared to other two AM fungal species [90].

Recently, it has also been tested a possible synergic effect of AM symbiosis and methyl jasmonate both to improve plant physiological performances under water stress [91] and to increase the production of SMs [92]. In particular, in *Trigonella foenum-graecum* (fenugreek), a leguminous plant frequently used in medicinal preparation for its content in trigonelline and diosgenin, it has been shown that AMF are more effective to stimulate the production of trigonelline, while the combination of methyl jasmonate and root symbiosis resulted a better elicitor for diosgenin production [92]. Furthermore, a recent study also highlighted a possible positive combined effect of bioregulators application (in this case, exogenous ethylene application by ethephon treatments) and AM symbiosis in stimulating trigonelline production in fenugreek [93]. Elicitation of SM production by the associated microbiota has been also reported in cannabis plants [94], showing a stimulation of cannabinoid compound concentrations and an antagonistic activity against invading pathogens and contaminating mycotoxigenic fungi [95].

Looking at the plant as an holobiont, attention has been also dedicated to evaluating the role of endophytic fungi in alterations of metabolite production and amounts of active compounds in medicinal plants. In addition, endophytic microbial communities associated with medicinal plants have a great potential as producers of bioactive compounds for agricultural, pharmaceutical, and other industrial applications [96,97]. Recently, SM profiles of endophytic fungi isolated from *Salvia abrotanoides* originated from three geographically distinct sites in Iran have been evaluated. Extracts from mycelia of these isolates revealed a wide spectrum of SMs, demonstrating that some of them (e.g., *Penicillium canescens*, *P. murcianum*, *Paraphoma radicina* and *Coniolariella hispanica*) produce cryptotanshinone, i.e., a main bioactive compound of *S. abrotanoides*, confirming that endophytic fungi play an important role in the production of plant bioactive metabolites [55].

Results so far obtained suggested that inoculation of plant species could represent a suitable tool to obtain higher amounts of metabolites for pharmaceutical and medicinal purposes, both using AMF and PGPB [45]. However, the impact can be different depending on the genotypes and on the cultivation conditions. In addition, although studies focused on the accumulation of SMs in inoculated plants have been intensified over the last decade, the mechanisms through which root-associated microorganisms can alter the production of SMs are not fully clarified yet.

## 3. Genetic and Genomic Approaches to Improve Specialized Metabolites

Given the relevance of different SMs in various functions in plants and for many food, pharmaceutical or industrial applications, there is considerable interest in research aimed at identifying key genes, developing innovative breeding strategies and biotechnological approaches towards modulating SM content.

### 3.1. Key Genes Identification

Successful strategies for pathway engineering rely on the careful and knowledge-based choice of the appropriate candidate genes that govern the output of the target pattern. Detailed knowledge is needed of all the enzymes implicated in the target pathway, their isoforms and the rate-limiting reactions for synthesis and catabolism, as well as of the spatial localization of metabolism [98,99,100]. This biochemical understanding of the core reactions needs to be conveyed to the identification of the genes coding for the key biosynthetic enzymes, the tailoring enzymes that catalyze the decorating reactions modifying the molecular backbones, as well as the regulating factors that orchestrate the functioning of the entire pathway.

Gene fusion and, to a minor extent, horizontal gene transfer, contributed to the evolution of metabolic networks, but the major drive for the diversification of the complexity of plant specialized metabolism is considered to be gene duplication, often of genes of the primary metabolism, resulting in dosage effects and neofunctionalization of paralogs [101]. In other cases, duplication itself could be interpreted as an adaptive trait, selected after a single gene had evolved a novel beneficial function at the expense of its ancestor [101].

Therefore, several gene isoforms can exist for any target gene, and the identification of the relevant one(s) for a given pathway is a challenging task, since minor changes in enzyme protein sequence may have dramatic effects on specificity and functions. Genome resequencing is increasingly contributing to the discovery of gene variants in a plethora of plant species, starting from earlier large work in species such as tomato, rice and *Arabidopsis thaliana* [102,103,104,105] to even more ambitious efforts, such as the 10KP Genome Sequencing Project, aimed at sequencing more than 10,000 genomes from plants and protists [106]. Along with massive analyses, resequencing of genotypes with peculiar characteristics may highlight structural differences in candidate metabolic genes, as was the case for genes involved in the biosynthesis, accumulation and decoration of phenolic compounds in two long shelf-life traditional varieties of tomato [107].

The vast and ever-increasing availability of genomic, transcriptomic, proteomic and metabolomic data for many plants, beyond model species and representative crops, has greatly accelerated and refined the process of identifying target genes controlling the plant metabolic diversity, and bioinformatics has become the most common starting point for the prediction of gene function. Tools such as plantiSMASH and PhytoClust allow automated analysis of genomic and transcriptomic data for the identification of candidate metabolic genes, also exploiting the clustering in close genomic regions that often occurs for this type of genes [108,109]. Analysis of co-expression networks across multiple conditions can be feasible also for non-sequenced species, and biologically relevant information can then be obtained by data integration with functional annotation databases such as Gene Ontology (GO) [110], the Kyoto Encyclopedia for Genes and Genomes (KEGG) [111], Mapman [112] or BioCyc [113]. Further understanding of gene functions and regulatory relationships can be inferred from gene-gene interactions, such as searching for targets of known transcription factors within the network of co-expressed genes, with several tools being available for gene interaction network analysis [114] or experimentally gained through protein–protein interactions detecting methods such as yeast-two-hybrid or affinity purification. Given the complexity of plant specialized metabolism and the high similarity between genes with different functions, the identified candidate genes need to be confirmed by forward or reverse genetics, or heterologous expression approaches.

Given all the obstacles that can hinder candidate gene(s) identification, however carefully and knowledge-based conducted, this approach may prove unsuccessful, also because the number of involved genes may still be beyond our prediction power. Epigenetic control and contribution from the organelle genomes add up further complexity to the definition of promising targets for metabolic engineering. Emblematic is a study on a quantitative trait locus (QTL) mapping analysis for carotenoid genes in a population of tomato introgression lines (ILs), which found that only 5 of 23 carotenoid biosynthetic genes co-segregated with QTLs for fruit color [115].

A relatively unbiased contribution to dissect the genetic control of plant metabolic diversity can come from genome-wide association studies (GWAS), which explore variation in breeding and natural populations and associate genetic variance to traits of interest, identifying specific QTLs. In cases where accurate metabolite evaluation methods were available, GWAS have been applied to plant metabolic studies (mGWAS) [116], and have proven to be more successful in identifying strong QTLs than GWAS applied to agronomic traits, possibly because of the large natural variation and high heritability of SMs accumulation [117]. Unlike GWAS for complex traits, mGWAS for accumulation of specialized metabolites tend to identify enzyme-coding genes rather than regulatory features, facilitating the prediction of candidate genes within genomic regions [117].

The development of low-coverage sequencing-based GWAS approaches [118] could expand the feasibility of using mGWAS in species lacking large genome sequencing resources, which could be the case for many plant species with peculiar metabolic features. Finally, the demonstration that the genetic architecture of plant metabolome can be highly conserved [119,120] indicates that comparative mGWAS data may provide information on candidate genes also between related species.

### 3.2. Advanced Breeding Strategies

#### 3.2.1. Molecular Assisted Breeding

Crop genetic improvement, the science of applying genetic and plant breeding principles and biotechnology to improve plants, represents one of the most effective strategies to develop new cultivars in which the modulated yield of a given specialized metabolite or phytochemical is stably fixed.

In the 1980s, the development of molecular (DNA) marker technology, and of the derived molecular genetic maps, has promoted crop improvement to rapidly evolve from classical breeding approaches based on phenotypic selection to more effective marker-assisted breeding (MAB). Compared to conventional breeding, MAB offers several important advantages as genotype screening allows more precise and rapid breeding schemes. Furthermore, MAB is particularly appropriate for integrating two or more genes or QTL into an elite genotype (gene pyramiding or gene stacking) [121].

Over the past decades, different types of molecular markers have become available for the development of molecular genetic maps and for the selection of the most favorable allele(s) [122]. However, the advent of next-generation sequencing technologies (NGS) along with powerful computational pipelines has enabled the development of more informative large-scale molecular markers, which allow faster and cheaper genotyping that revolutionized plant breeding [121,122,123].

The flourishing developments in omics technologies at multiple layers including but not limited to genomes, transcriptomes, epigenomes, epitranscriptomes, proteomes and metabolomes are further enhancing crop breeding programs [124].

The approaches used for the identification of the genetic determinants of the phytochemical component of plants can be either reverse genetics or forward genetics. Within the past 20 years, targeted reverse genetics approaches have played a major role in investigating plant metabolism [116]. By contrast, top-down approaches have recently been pursued to explore the wide natural genetic diversity for specialized metabolism harbored in interspecific backcross inbred lines (BILs), ILs and, more recently, GWAS, which have been demonstrated to be effective tools to gain further insights into the regulation of plant primary and specialized metabolism [116,125,126,127,128].

Metabolomics-assisted breeding by means of species-wide comparisons represents an emerging approach for metabolic crop improvement [129,130]. More recently, for tomato, Zhu et al. [131] developed a Multi-Omics Database, consisting of metabolomics, vari-omics (mutation data), and transcriptomics data in addition to genomic data, which represents a rich resource for plant metabolic biology and breeding.

As regards the use of natural variability as a rich source of potentially valuable genes/alleles for breeding programs that aim to modulate the content of SMs in plants, the Solanaceae family represents a good reference system. Specifically, in tomato, two independent studies, based on a mutant inbred line [132] or an interspecific *Solanum chmielewskii* IL population [133], demonstrated that the colorless-peel *y* mutant on chromosome 1 is controlled by a *SlMYB12* regulated transcriptional network, which orchestrates the accumulation of yellow-colored flavonoid (naringenin chalcone) in the fruit epidermis (peels from pink fruit are colorless due to the absence of flavonoid). Subsequently, a GWAS using 231 tomato accessions, with known phenotypes, allowed the identification of the causative variant, with three recessive alleles underlying the *y* phenotype, which represent useful markers for pink tomato breeding [103]. The same *S. chmielewskii* IL population was used to identify genomic regions underlying the production of tomato fruit semi-polar specialized metabolites, including alkaloids and phenylpropanoids [134]. The integration of biochemical pathway knowledge and genomic information allowed the identification of several candidate genes, which could be used in breeding programs. Moreover, in eggplant (*Solanum melongena*), an interspecific mapping population was used to map candidate genes for fruit chlorogenic acid content [135,136]. This was assisted by the use of synteny of the orthologous genes in tomato, the solanaceous species with the broadest genetic and genomic toolkit. In peppers (*Capsicum* spp.), the combined use of genome-based QTL mapping and GWAS has proven to be a powerful approach to identify candidate genes associated with capsaicinoid content, which was not easily achieved in previous studies using low-density genetic maps [137]. The obtained results have also confirmed the minor effects of each locus and the epistatic effects between QTLs, suggesting that multiple markers should be used together for marker-assisted selection.

When the candidate genes have been identified, and functionally verified, specific markers can be developed for a more efficient MAB of improved cultivars. For instance, in pepper, marker-assisted selection has been pursued to develop a new fresh pepper cultivar containing capsinoids, low-pungent capsaicinoid analogs. For this purpose, PCR-based markers associated with *p-AMT* and *Pun1* genes were developed and used to obtain a new cultivar with desirable traits [138].

However, despite all the mQTL identified for SMs, only a few candidate genes have been functionally validated so far [139], and only a limited number of molecular markers have been successfully used in MAB programs [140]. The reasons for the gap that still exists between the information about the genes and QTL that underpin plant phenotypes and the integration of this information into applied plant improvement are manifold [121,141]. However, this scenario should be reversed by a wider application of omics data, which promise to accelerate QTL cloning [142], along with improved tools for marker-assisted selection (MAS) (i.e., reliable markers and robust QTL) and enhanced strategies for integrating these tools into breeding programs effectively, without associated genetic drag [141]. In parallel to developments in NGS technologies, newly designed multi parent mapping populations, such as nested association mapping (NAM) and multi- parent advanced generation inter-cross (MAGIC), have also been developed, which are suitable for joint-linkage and association mapping and which significantly improve the power and efficiency of GWAS [117,121]. Furthermore, NGS-based approaches, including sequencing-based mapping (SbM), can be used in combination with bulked segregant analysis (BSA), and similar strategies, to help speed the identification of candidate genes [121]. For example, the QTL-Seq approach involves the use of whole genome resequencing (WGRS) on bulked DNA samples from the phenotypic extremes of a population of recombinant inbred lines (RILs) or F2 individuals derived from inter-varietal crosses [121,139].

Therefore, NGS plays a key role in a genomics-assisted breeding pipeline, as it enhances the speed and precision of trait mapping to identify genes and QTL that become the targets of MAS. Furthermore, NGS allows calculating the genomic estimated breeding values (GEBVs) based on genome-wide information that predict the breeding value of individuals in a breeding population using genomic selection (GS) [121].

Finally, new breeding technologies (NBT), such as genome editing, have complemented molecular breeding and transgenesis for precision breeding, paving the way to the recently proposed “5G breeding approach”, which ideally should integrate Genome assembly, Germplasm characterization, Gene function identification, Genomic breeding (GB) and Gene editing (GE) [124].

#### 3.2.2. Genetic Modification of Crop Plants

Metabolic engineering could be particularly useful whenever traditional or MAB approaches are hindered by low variability in natural and breeding populations, and limited -omic tools. It includes overexpression and/or competition with the target pathway, overcoming rate-limiting steps, stopping the catabolism pathway of the desired product and/or inhibiting other pathways with the final aim of optimizing the production of target specialized compounds.

Pathway engineering programs make use of gene cloning technologies and different gene transfer technologies such as *Agrobacterium*-based transformation or alternative methods (e.g., particle bombardment and electroporation), to ensure transient or stable DNA introduction into host plant cells to manipulate plant metabolism [143,144]. Although numerous species are recalcitrant to different transformation methods, to date, ample literature is available with examples that demonstrate how approaches based on insertion or deletion of key genes from the core metabolic pathway or on ectopic expression of transcription factors (TFs) that control biosynthetic pathways (see more details in Section 3.3) have been employed to modulate levels and identity of specialized compounds in several plant species [145]. Several efforts have been dedicated to the modification of the phenylpropanoids pathway in crops [146,147]. As an example, the core flavonoids metabolic pathway has been addressed towards improving these relevant compounds in *S. lycopersicum* [147,148]. The first attempts to induce flavonoid production in tomato started in the early 2000s with the overexpression of different structural genes of the biosynthetic pathway. In tomato, flavonoids (i.e., naringenin chalcone and rutin) are present mainly in the peel and only traces can be detected in the flesh. Ectopic expression of single (chalcone isomerase, *CHI*) or multiple (chalcone synthase, *CHI*, flavanone 3-hydroxylase and flavonol synthase) structural genes successfully enhanced the levels of flavonols (i.e., quercetin- and kaempferol- glycosides) in the peel [98,149,150] and flesh [149] of tomato fruit. Although an increase in the accumulation of different phenylpropanoids was observed, contributing to increase the total antioxidant activity, no anthocyanin synthesis was registered [98,146]. Recently, co-expression of the onion chalcone isomerase in *Delila bHLH* and *Rosea 1 R2R3-MYB*-expressing purple tomato (see more details in Section 3.3) converted the flavonoid flux to flavonol as well as further increased the content of anthocyanins [151], highlighting that combined strategies based on the modulation of structural genes and TFs that control this biosynthetic pathway are highly effective.

In the last decade, plant in vitro systems such as culture of organs (e.g., hairy roots) and cells confirmed their several advantages as “green cell factories” to produce specialized metabolites, as compared to both transgenic plants and heterologous microbial systems (e.g., yeast and bacteria) [152] (see more details in Section 4.1). In particular, Hairy Root Culture (HRC) has been widely explored for producing valuable specialized metabolites [153]. HRCs can be obtained by transformation of plants with *A. rhizogenes* that introduces the genes *rolA*, *rolB*, and *rolC* in the host plant genome by the root-inducing (Ri) plasmid [154]. Transformed roots are characterized by higher growth rate, genetic stability and can be propagated in growth regulators-free media also in large-scale bioreactors [155,156]. Singh and collaborators have developed a protocol where *A. rhizogenes* was used to induce hairy roots in the tomato explants and demonstrated that they efficiently accumulate several health-promoting compounds such as rutin, quercetin and kaempferol [157]. A recent study describes protocols to establish stable hairy root lines from leaf explants of radish. The produced transgenic hairy roots showed increased production of flavonoids (quercetin) [158]. Strategies based on overexpression of a R2R3-MYB-type TF (*VvMYB15*) in *V. vinifera* hairy roots allowed to get an increased production of stilbenes, a small group of phenylpropanoid compounds with a role in plant defense but also with pharmacological properties in the prevention/protection from cardiovascular and neurodegenerative diseases, cancer and diabetes [159].

Both *Agrobacterium* and particle bombardment-based transformation have been effective for an array of plant species; however, several challenges such as limited host range of *Agrobacterium*, stability of the compounds produced in hairy roots and low regeneration after bombardment limit their use in many crops [160,161]. In recent years, a new transformation approach based on various nanomaterials like carbon nanotubes or magnetic nanoparticles can bypass the barrier of the cell wall and transfer their cargo including nucleic acids into plants in a transient or stable manner [162,163]. Thus, nanoparticle-mediated gene transformation could represent a novel gene delivery tool in plant; however, its implementation is still at an early stage.

#### 3.2.3. New Breeding Techniques (NBTs)

NBTs have the potential to change the pace and course of crop biotechnology by overcoming most of the limitations imposed by conventional genetic engineering techniques, as they allow fast and precise targeted genome modifications, in some cases indistinguishable from naturally occurring mutations, without leaving any foreign DNA. NBT comprise the use of zinc finger nucleases (ZFNs), transcriptional activator-like effector nucleases (TALENs) and clustered regularly interspaced short palindromic repeat (CRISPR)/CRISPR-associated nuclease 9 (Cas9) system [164]. However, due to its high flexibility, versatility, specificity, efficiency and low costs, CRISPR/Cas9-based technology rapidly evolved and became dominant in plant genome editing [165]. Besides the well-known Cas9, new Cas nucleases have been identified and/or improved to generate CRISPR/Cas twins with new properties. For example, Cas12a/Cpf1, xCAS9 and Cas9-NG are able to recognize a wider range of PAMs, while Cas12a/Cpf1 cleaves the target DNA generating staggered ends [166,167,168]. Furthermore, the fusion of a catalytically inactive Cas9 (Cas9 variants, dCas9 or Cas9 nickase) to a cytosine or adenosine deaminase domain allows the conversion of C:G to T:A, and A:T to G:C, respectively [169,170]. These base editing (BE) systems are expected to be further implemented thanks to the recent discovery of “prime editing” that allows targeted insertions, deletions, all 12 possible base-to-base conversions and combinations thereof at genomic targets without any requirement of double strand breaks or donor DNA templates [171].

The very fast development and expansion of genome editing tools have opened new possibilities to manipulate and modulate gene expression acting at several levels including transcription, mRNA processing and mRNA translation. All these processes are under the control of a series of *cis*-regulatory elements, which can be modified by genome editing technologies. For this purpose, tool kits have been developed, and their efficacy has been proven, in which dCas9 is fused to effector domains, such as transcriptional activators and repressors, to modulate expression of target genes [166,172,173].

CRISPR/Cas9 technology has been mainly used to investigate the plant biosynthetic potential through switching off the competing biosynthetic pathways and shifting of the metabolite flux toward the production of the target compounds. For example, a considerable decrease in thebaine, codeine, noscapine and papaverine levels in stem together with a dramatic reduction in the S-reticuline and laudanosine has been achieved by editing the *4′OMT2* gene, which regulates the biosynthetic pathway of benzylisoquinoline alkaloids in *Papaver somniferum* [174]. The authors also reported that the edited plants produced a novel uncharacterized alkaloid, showing the potential of the CRISPR system in the production of new SMs. CRISPR/Cas9 approach was also used to edit the rosmarinic acid synthase (*SmRAS*) gene in *Salvia miltiorrhiza* [175]. This modification led to a reduction of phenolic acids content such as rosmarinic acid together with an increase in its precursor 3,4-dihydroxy phenyl lactic acid, in the edited hairy root lines, especially in the homozygous. In another study in *S. miltiorrhiza*, CRISPR/Cas9 was used to knock out the *SmCPS1* gene coding for a diterpene synthase, involved in the synthesis of tanshinones, with the aim to evaluate the possibility to promote the accumulation of the substrate for taxol synthesis, as tanshinones and taxol share the same precursor (Geranylgeranyl Pyrophosphate) [176]. A number of studies have focused on the manipulation of anthocyanin and flavonoid biosynthetic pathways. Flower color modification using the CRISPR/Cas9 system was successfully achieved in *Torenia fournieri* L. flowers by editing a flavanone 3-hydroxylase (*F3H*) gene, while flower shapes and colors were altered in *Ipomoea nil* by editing the dihydroflavonol-4-reductase-B (*DFR-B*) gene [177,178].

When dealing with specialized metabolism, one of the most desired objectives is to redirect the substrates stream by removing competing pathways. A nice example of the use of NBTs for this purpose could be the multiple editing of the squalene synthase (*SQS*), β-farnesene synthase (*BFS*) and β-caryophyllene synthase (*CPS*) genes encoding enzymes at branching points for the synthesis of artemisinin, an antimalarial drug, in *A. annua*. In fact, Lv et al. [179] showed that artemisinin increased in anti-SQS, anti-BFS and anti-CPS transgenic plants.

Together with key biosynthetic structural genes, interesting candidates for the application of NBTs to modulate specialized metabolisms are represented by TFs (see next paragraph). Hypothetically, specific biosynthetic pathways can be constitutively switched on mutating negative regulators or using CRISPR/Cas fusions with activators directed towards key structural genes or positive regulators [180,181]. An example is the CRISPR-Cas9 SunTag systems consisting of two modules: dCas9 fused to tandem GCN4 peptide repeats and a single chain variable fragment (scFv) GCN4 antibody fused to superfolder-GFP (sfGFP) and VP64. In this way, multiple copies of the VP64 transcriptional activator associate with the GCN4 repeats and are recruited to a specific locus via dCas9/guide RNAs [182].

Although NBTs can be used theoretically in any plant and crop species for which the genomic sequences are accessible, several technical restrictions still exist. For instance, the most important is the recalcitrance to transformation of many medicinal plants harboring pharmaceutical bioactive components and other valuable metabolites.

### 3.3. Regulation of Specialized Metabolism and Modulation of Transcription Factors

Recently, the characterization of enzymes involved in the production of specialized metabolites has advanced enormously, while knowledge of pathway regulation is still somewhat limited [183]. In such a dynamic context as the one of plant metabolism, the subject of regulation can be addressed from many different perspectives. To maximize fitness and to resolve the cost-benefit paradigm, regulatory networks evolved in the control of perception of external and internal cues, as well as in controlling cellular processes such as epigenetic landscaping, transcription, translation, and allosteric control of the resulting enzymes and regulators [184]. For this reason, a comprehensive analysis of the regulation of specialized metabolism should always be addressed from the perspective of combinatorial control among many different pathways, in a broader attempt to harmonize ecological, cellular and molecular data.

Aware of all the above-mentioned regulatory networks, it is clear that research has largely focused on the role of TFs, which play pivotal roles in regulating genes involved in all aspects of plant development, including specialized metabolism. TFs are regulatory proteins that modulate the expression of genes, and the set of genes regulated by a specific TF is referred to as a regulon. TFs bind—alone or as part of multimeric complexes—specific short sequences (*cis*-regulatory elements) in the regulatory regions of target genes, promoting or blocking the recruitment of the transcriptional machinery [185]. Mutations and neo-functionalization of metabolic genes are largely responsible for the increase in chemo-diversity among metabolites; however, there are limits to the extent that an enzyme can be modified without affecting its stability. For this reason, it has been proposed that TFs play a priming role in the recruitment of metabolic genes into regulons—as a consequence of the emergence of cognate *cis*-regulatory elements, which might largely fuel the diversification of specialized metabolic pathways [186,187]. While plant regulons often consist of genes scattered through the genome in non-contiguous loci, there is evidence that biosynthetic gene clusters (BGCs) and regulatory gene cluster (RGCs) might be recurrent for pathways of specialized metabolism; this is the case for steroidal glycoalkaloid genes organized in a BGC in tomato, all controlled by a RGC of AP2/ERF TFs resulting from tandem repeats of ancestral genes [184,188]. All major families of TFs have been described as regulators of plant specialized metabolism. Due to the combinatory nature of metabolic networks, closely related enzymes can be recruited in different regulons and TFs of different families contribute (synergistically or antagonistically) in the regulation of metabolic pathways [189].

In the last decades, the modulation of TFs expression through genetic engineering approaches has emerged as a highly powerful tool to improve specialized metabolism for specific purposes, such as the improvement of the nutritional quality of different crops. In theory, this modulation ensures a relatively coordinated and almost “stoichiometric” production of enzymes, allowing for optimized biosynthesis [190]. Different strategies may be used: from the traditional overexpression of the TF under the control of a constitutive promoter to a tissue specific expression or induction of point mutations through gene editing techniques. However, such approaches are not always a sure hit, as a single TF is not always sufficient to regulate an entire whole biosynthetic pathway, which usually requires regulatory networks consisting of multiple TFs [191].

The functional characterization of TFs involved in the control of anthocyanins metabolism is a good example of how the study of transcriptional regulation provides the basis to fine tuning of specialized metabolic pathways. Anthocyanins are a large family of phenolic compounds widespread in flowers, fruits and vegetables that protect plants from a wide variety of biotic and abiotic stimuli and attract pollinating insects and seed-spreading animals. They are a valuable target in food and crop biofortification as their antioxidant and anti-inflammatory actions are beneficial to prevent and control diseases, such as cardiovascular disorders, diabetes, obesity, cancer and neurodegenerative diseases [192]. Moreover, anthocyanins (and other metabolites) contribute to color in plants, an aspect that strongly drives consumers’ preferences [193].

Anthocyanin accumulation in plants is modulated by the WD-repeat/bHLH/MYB complex, that positively regulates dihydroflavonol 4-reductase, anthocyanin synthase and glucosyltransferase gene expression [194]. This molecular mechanism was proven to be conserved across many different species via orthologous TFs, being crucial in the determination of color of flowers and fruits in apple, grape and oranges [189]. An indirect tinkering of the modulation of this transcriptional control is normally used in standard agriculture and horticulture procedures. For example, as light and UV-B have major roles in activating the expression of TFs that positively regulate anthocyanin biosynthesis, light exclusion through the practice of bagging apples prevents anthocyanin accumulation. Subsequent removal of the bag prior to harvest results in a rapid and uniform accumulation of anthocyanins [193].

Genetic engineering could help obtaining further benefits from the tuning of this regulatory mechanism; this was proven by the molecular strategies used to increase anthocyanins in tomato fruits, recently summarized [195]. Cultivated tomatoes do not accumulate anthocyanins in their fruits, although biosynthetic genes are present in their genome, and some wild tomato species produce green fruits that, upon suitable light conditions, can accumulate anthocyanins in the peel [196]. Hence, researchers moved to the TF strategy with the overexpression in tomato of two maize TFs, belonging to the bHLH and R2R3-MYB families, known to regulate anthocyanin biosynthesis. Moreover, this approach turned out to be unsuccessful, due to the lack of activation of one of the tomato anthocyanin synthesis structural genes [197]. In 2008, another attempt was successful: Butelli and colleagues [198] overexpressed the *Delila bHLH* and the *Rosea 1 R2R3-MYB* genes from *Anthirrinum majus* under the control of the fruit-specific E8 promoter, obtaining tomato fruits accumulating anthocyanins at a concentration of 2 mg g^−1^, similar levels as in blueberries and blackberries. The purple tomatoes showed a protective effect against cancer progression when used to feed cancer-susceptible Trp-53-/-knockout mice [198]. The purple tomato line was then crossed with another line expressing the *AtMYB12* gene, known to regulate flavonol biosynthesis in *A. thaliana*, under the fruit-specific E8 promoter. The Indigo tomato was obtained, characterized by a strong increase in chlorogenic acids, flavonols and anthocyanins, with an increased total antioxidant activity [199]. Finally, the *V. vinifera* stilbene synthase gene was overexpressed in the Indigo line, and the obtained Bronze tomatoes, characterized by increased flavonols, anthocyanins and stilbenoids, alleviated inflammation bowel disease in mice [200].

These examples and the ever-growing wealth of data about the role of TFs in specialized metabolism show that artificial modulation of transcriptional regulation could be one of the most promising strategies towards biofortification. Understanding the structural characteristics of these genes (and their encoded proteins) explains how TFs are regulated at a transcriptional (auto-/cross-regulations), post-transcriptional (alternative splicing, miRNA) and post-translational levels (interactions, localization and modifications), unveiling new strategies for the rational engineering of the metabolic pathways that can be implemented through transgenic technologies, genome editing and and/or marker assisted breeding.

### 3.4. Metabolism of Plant Glandular Trichomes: A Target for Molecular Breeding and Biotechnological Approaches

Given the bioactive nature of SMs, plants have developed specific structures for their accumulation, in order to safely produce and store these compounds while avoiding their possible adverse effects. Considering the vast repertoire of metabolites, tissues accumulation, cell type production, storage and release of these specialized molecules, in this paragraph, we will focus on metabolite production in glandular trichomes. Trichomes are a dedicated biosynthetic and storage structure consisting in epidermal extensions occurring on the surface of the aerial part of plants. They are widely distributed in the plant kingdom and can be distinguished in non-glandular and glandular trichomes. Non-glandular trichomes are widely diffused in angiosperms and gymnosperms down to lycophytes, ferns and bryophytes. The model species *A. thaliana* possesses non-glandular trichomes, whereas all the essential oils-producing species within Lamiaceae, such as mint, sage, rosemary, basil, but also Asteraceae and Solanaceae, possess glandular trichomes. Glandular trichomes are able to produce, store and often secrete exudates carrying a terrific plethora of chemo-diverse molecules, such as essential oils and oleoresins, phenols, glycerids and very complex terpenes. Outstanding examples of high economic value are the anti-malarial artemisinin in *Artemisia annua* [201], psychoactive cannabinoids in *Cannabis sativa* [202], and the psychotropic molecule salvinorin A in *Salvia divinorum* [203], but also acylsugars that are glycolipids consisting of a sugar core linked to straight or branched acylic chains [204]. To biosynthesize these complex molecules, plants require an incredible energetic effort. The trade-off for the producing plants resides in the ecological role of these molecules that allows increased fitness and higher capability to survive in hostile environments.

Since the first studies on mint secretory glands and the identification of key enzymatic steps in menthol biosynthesis [205], scientific interest toward biochemistry of trichomes, as biofactories of valuable molecules, has enormously increased. This has been particularly remarkable in the last ten years, when the emergence of high-throughput sequencing techniques has allowed deepening the knowledge on the genes responsible for metabolic diversity. In parallel, efficient isolation techniques by laser microdissection pressure catapulting (LMPC) [206] have allowed the separation and enrichment of specific cell types, such as multicellular glandular trichomes, thus targeting chemical, transcriptional and biosynthetic characterizations only toward glandular specialized metabolites. The combination of these techniques with advanced mass spectrometry technologies, UPLC-MS/MS, LC-TOF-MS, NMR, or even mass spectrometry imaging (MSI) has allowed direct profiling and imaging of the glandular trichomes metabolism. In particular, imaging studies resulted very useful for detecting shape and development of glandular trichomes in cultivated and wild tomato species [207]. Further, Balcke et al. [208] showed that the wild species *Solanum habrochaites* has trichomes with different shape and higher metabolic capacity than the cultivated *S. lycopersicum*, both characters being responsible for an almost 100-times higher terpenoids accumulation than in cultivated tomato. In wild tomato species, the high density of a specific type of trichomes is also responsible for the high production of acylsugars [204]. The high protective role of wild tomato acylsugars is linked to their toxic and sticky nature, which confers the ability to trap and immobilize insects. Moreover, these molecules are also an efficient protection from fungal infections [209,210].

Cultivated and wild tomato species produce structural diverse acylsugars, and this diversification is associated with gene duplication and functional diversification of related biosynthetic genes. To provide plants with improved resistance, several breeding strategies have been designed in order to make cultivated tomato able to produce “wild” acylsugars. QTL mapping in recombinant populations has allowed to identify and introgress key genes in cultivated tomato lines [211,212,213]. As well, major genetic factors behind differences in trichome morphology have been identified by QTL analyses [214]. In parallel, overexpression or gene silencing biotechnological strategies have been used to boost the accumulation of glandular specialized metabolites, with the aim to increase plant protection and yield of bioactive molecules. As an example, R2R3 MYB-dependent auxin signaling pathway TFs, known to participate in hormonally regulated II, V and VI type trichomes formation in tomato leaves, have been used to successfully modulate trichome production [215], showing that increased trichomes density improved tomato tolerance to spider mites. Besides *Solanum* species, which are considered a model for trichome studies for their anti-herbivory potential, the overexpression of *AaMYB1* and its orthologue *AtMYB61* TFs was effective in increasing terpene metabolism and trichome development in *A. annua* and *A. thaliana* [216]. Implementation of metabolic engineering strategies is already taking advantage of the recent advances in CRISPR/CAS9 technologies, which result particularly promising for precision breeding of trichome complex traits. As an example, Campbell et al. [217] by combining classical genetic approaches (genetic mapping, array comparative genomic hybridization and whole genome sequencing) identified a soybean orthologue of *A. thaliana* CPR5, a necessary gene for proper trichome growth and development, and used a CRISPR/Cas9 functional approach to induce mutations that resulted in an altered trichome phenotype.

## 4. Plant Suspension Cell Cultures as Bio-Factories of Natural Compounds

### 4.1. Specialized Metabolism in Undifferentiated Cells: Constraints and Opportunities

Plant cell culture is a technology for cultivating cells and tissues under strictly controlled environmental conditions. Plant cultured cells, being undifferentiated, have the potential to express the full genetic machinery coded in the nucleus and thus are considered totipotent. Nevertheless, they are able to produce SMs, characteristic of original plants. Cultured cells, as callus or suspension, of many plant species have been established starting from differentiated tissues, such as leaves, stems or roots and proved to produce specific metabolites in axenic conditions. Particularly, suspension cell cultures offer the advantage of scaling up using bioreactors, which allow the cultivation of large-scale cell volumes and consequently the controlled production of increased metabolite amounts, independently from environmental conditions such as climate or soil [218]. For this reason, in vitro production appeared as a valid alternative to the use of whole plants, overcoming the limitations of adverse conditions occurring in the field due to climatic changes and/or pathogen attacks.

It is also known that many metabolites are present in plant tissues at very low concentrations, and the availability of a controlled production system such as suspension cell cultures producing specific phytochemicals is far desirable. The very important advantages of in vitro plant cultured cells are the fast growth and the ability to accumulate large amounts of uniform biomass in a short time period [218,219]. This is especially true to produce rare bioactive compounds, which in plants are usually found in low amounts, such as resveratrol, paclitaxel or terpenoids, whose isolation and purification requires the processing of large quantities of plant biomass [220,221,222]. Moreover, plant cultured cells offer a reliable and powerful production platform for continuous supply of contamination-free, phytochemically uniform biomass of diverse plant species such as aromatic, medicinal and even rare and threatened plants. The opportunity to obtain natural molecules by using an environmentally sustainable biosynthetic platform made plant cell culture technology exceptionally attractive for the production of active ingredients for high added value “green” cosmetic formulations [223].

Plant cultured cells are usually obtained by inducing dedifferentiation of already differentiated cells from adult specialized tissues. Because of their origin from differentiated cells, they could inherit some epigenetic modifications, characteristic of the tissues of origin, and thus, they could be very heterogeneous in their biosynthetic and growth properties. This evidence made it possible to generate almost unlimited numbers of plant cell lines with unique phytochemical profiles and growth characteristics even from the same plant of origin. On the other hand, epigenetic changes can also occur during in vitro maintenance, likely being the reason for the yield variations, sometimes observed in selected highly producing cell lines. Such instability may be a limitation that should be considered while using plant cultured cells as bio-factories of specific phytochemicals [224].

As previously stated, many phytochemicals, especially terpenoids, are biosynthesized in plants in specialized structures such as glandular trichomes [225]; nevertheless, in vitro cell cultures have the capacity of synthesizing such compounds. This is the case of the sesquiterpene lactone artemisinin produced by suspension cell cultures of *A. annua* [226]. It is interesting that *Artemisia* artemisinin “secretion” by the apical and subapical cells is mirrored in *Artemisia* cultured cells by the exudation of artemisinin to the culture medium [226,227]. This feature can be very useful to optimize an in vitro production system since it is easier to recover the desired compounds from the spent culture medium.

### 4.2. Valuable Chemical Tools to Improve Axenic Production of Bioactive Metabolites

Plants exhibit a wide array of constitutive and inducible defense strategies against biotic and abiotic stresses. Defense responses in plants are generally triggered by elicitors which act as chemical signals. In plant cell cultures, the exogenous supply of chemical elicitors including cellular signaling compounds such as jasmonic acid (JA), salicylic acid (SA), ethylene (ET), nitric oxide (NO) or synthetic chemicals mirrors an under-stress environment. The objective of adding elicitors to in vitro cultures is to misguide the cells for a possible biotic/abiotic attack, mimicking the external environment like natural conditions under stress [228]. Enhanced yield of specific phytochemicals, higher gene expression and discovery of new biomolecules are the effects of elicitor treatments that are detected as changed genetic and biochemical activities in the cellular background [229,230]. By the application of elicitors, both qualitative and quantitative modulations in the content of specialized metabolites can be obtained. Chemical elicitors such as JA, methyl jasmonate (MeJA), 2-hydroxyl ethyl jasmonate, SA, acetyl salicylic acid (ASA), trifluro ethyl salicylic acid, ET, NO, sodium nitropruside (SNP), ethrel or ethephon (Ethe), cyclodextrins (CD) and many more have been employed for secondary metabolite in vitro manipulation [230].

JA and its related signal molecules such as MeJA were extensively used for elicitation studies involving plant cell cultures (Table 1). Keeping in view the enormous literature on the use of jasmonates in elicitation studies, a few latest examples were cited herein involving plant suspension cell cultures. The concentration of elicitors could be vital irrespective of different plant species used for elicitation experiments. Different studies indicated concentration of jasmonates ranging from 5.0 to 500 µM for different plant culture systems, with concentration of 100 µM MeJA being used in most experiments involving several plants and culture systems (Table 1).

Similar to other chemical elicitors, SA and salicylates are used less than jasmonates for elicitation studies in plant cell cultures. These molecules may also act through modulation of biosynthetic enzymes, such as phenylalanine ammonia-lyase (PAL), tyrosine aminotransferase (TAT), superoxide dismutase (SOD), catalase (CAT) and peroxidase (POD) [231]. Table 1 lists selected salicylate elicitation involving in vitro plant cell culture systems.

ET is not a widespread chemical elicitor used for SMs yield enhancement due its positive as well as negative effects and its utilization as an elicitor is much more of academic than practical importance. Combined treatment of ET with other elicitors was found beneficial for enhanced production of SMs indicating its interactive role. Indeed, it was found that, independently of the use of elicitors alone or in combinations, the cell culture system was also critical in determining helpful effects on accumulation of metabolites (i.e., alkaloids).

The study on the role of NO for metabolites production in plant cell cultures was restricted only to a few researches. Enhanced taxol production (11% more than control cultures) was obtained with 20 µM SNP treatment using immobilized cell cultures of *Taxus cuspidata* [232].

CD are cyclic oligosaccharides, known as drug carriers for their ability to complex, thus solubilizing, various compounds. Nevertheless, they were reported to effectively improve the production of bioactive compounds in plant cell cultures [233].

Carbohydrates and derivatives purified from complex crude fungus or yeast extracts have been utilized as elicitors of plant cell culture. such as chitin, a long-chain polysaccharide of -(1-4)-N-acetyl-D-glucosamine units synthesized by fungi and yeast, in which it is a characteristic cell wall component [234].

**Table 1:** Specialized metabolites enhanced by the application of cellular signaling compounds in plant suspension cell cultures.

Plant Species	Elicitor	Metabolites	References
*Capsicum chinense*	SA, CaI	Capsaicin	[235]
*Papaver somniferum*	SA, H_2_O_2_, CO_2_	Sanguinarine	[236]
*Crocus sativus*	SA	Crocin	[237]
*Linum album*	SA	Podophyllotoxin	[238]
*Cayratia trifolia*	SA, MeJA, Ethe	Stilbenes	[239]
*Artemisia absinthium*	MeJA, JA	Flavonoids, phenolics	[240]
*Gymnema sylvestre*	MeJA, SA	Gymnemic acid	[241]
*Papaver bracteatum*	MeJA, US	Thebaine	[242]
*Arnebia euchroma*	MeJA	Shikonin	[243]
*Genista tinctoria*	MeJA, DMSO	Isoflavones	[244]
*Eryngium planum* L.	MeJA	Rosmarinic acid CGA, CFA	[245]
*Vitis vinifera*	MeJA, CD MeJA, Coro	Trans resveratrol, Stilbenes, Anthocyanin	[246] [247] [248]
*Fagopyrum esculentum*	MeJA, SA	D-chiro-inositol	[249]
*Artemisia annua*	MeJA, miconazole MeJA, CD	Artemisinin	[226] [227]
*Capsicum chinense*	MeJA, SA	Vanillin, capsaicinoid	[250]
*Catharanthus roseus*	MeJA, SA, ET	Vindoline	[251]
*Erythrina Americana*	JA	Erysodine	[252]
*Taxus canadensis*	MeJA, ET	Taxanes	[253]
*Hypericum perforatum*	NO	Hypericins	[254]
*Taxus chinensis*	JA, MeJA, TFEJA	Tax-C	[255]
*Cupressus lusitanica*	MeJA, ET	β-thujaplicin	[256]
*Vitis vinifera*	Chitosan/chitin	Stilbenes, trans-resveratrol	[257]
*Taxus chinensis*	Chitosan/and chitin	Paclitaxel	[258]

CFA, caffeic acid; CaI, calcium ionophore; CD, cyclodextrins; CGA, chlorogenic acid; CO_2_, carbon dioxide; Coro, coronatine; DCCD, N, N0-dicyclohexylcarbodiimide; DMSO, dimethyl sulfoxide; ET, ethylene; Ethe, ethrel/ethephon; H_2_O_2_, hydrogen peroxide; HEJ, 2-hydroxyethyl jasmonate; HM, heavy metals; JA, jasmonic acid; MeJA, methyl jasmonate; NO, nitric oxide PFPJA, pentafluoropropyl jasmonate; SA, salicylic acid; SNP, sodium nitroprusside; TFEJA, trifluoroethyl jasmonate; US, ultrasound.

## 5. Conclusions and Future Perspectives

Even if not exhaustive, due to the increasing interest of the scientists on SMs, the examined literature supports the hypothesis that the biosynthesis of plant secondary metabolites (also called specialized metabolites) in many Mediterranean crops is stimulated by various environmental constraints (salinity, drought, temperature, etc.) and that these compounds play a significant role in plant adaptation to environmental stresses. The interactions with other organisms, such as soil fungi and bacteria, can also lead to a different accumulation of diverse SM classes both at local and systemic level (Figure 1). Most of SM properties are associated with their functional groups exerting an antioxidant activity against oxidative damage that is usually the result of excessive generation of reactive oxygen species (ROS) due to environmental stress. It is also documented in the literature that plant SMs are important candidates for human nutrition. Biotic and abiotic factors that influence production of plant SMs should be further investigated with the aim to set up crop management strategies to improve the potential of plants for SM production in open fields or in confined environments and thus their nutritional quality. Environmental stresses experienced by many important crops in the Mediterranean environment may constitute not only a constraint for their production but also a tool for their exploitation and for improving the synthesis of different SMs of interest in vitro as well in vivo.

Moreover, research is needed to completely understand the regulatory proteins and genes involved in the biosynthesis of plant SMs so that these may be manipulated for improving plant tolerance to environmental stresses.
